# A Clinical Approach for the Removal of a Large Antral Pseudocyst with Simultaneous Maxillary Sinus Augmentation: A Case Series

**DOI:** 10.3390/medicina60050838

**Published:** 2024-05-20

**Authors:** Won-Bae Park, Jina Shin, Seungil Shin, Ji-Youn Hong

**Affiliations:** 1 Private Practice in Periodontics and Implant Dentistry, Seoul 02771, Republic of Korea; wbpdds@naver.com; 2 Department of Dentistry, Graduate School, Kyung Hee University, Seoul 02447, Republic of Korea; jina_s7528@naver.com; 3 Department of Periodontology, College of Dentistry, Kyung Hee University Dental Hospital, Kyung Hee University, Seoul 02447, Republic of Korea; shin.dmd@khu.ac.kr

**Keywords:** antral pseudocyst, dental implant, lateral sinus window, maxillary sinus floor augmentation, Schneiderian membrane

## Abstract

For a large benign lesion within the maxillary sinus, such as an antral pseudocyst, maxillary sinus floor augmentation is more commonly performed using a two-stage approach. This involves first removing the lesion, and then, re-entry following several months of healing. In this case series, we described the “one-bony-window” approach, which is a technical surgical modification of the previous one-stage approach, for simultaneous cyst removal and maxillary sinus floor augmentation. Four patients with large maxillary antral pseudocysts were included. The “one-bony-window” approach involves the preparation of a large window opening of approximately 15 mm × 20 mm at the lateral wall. A mesiodistally extended intentional perforation was made in the upper part of the exposed membrane to enhance the access for instrumentation. The antral pseudocyst was removed in its entirety without being deformed to prevent rupture or leakage of the cystic contents. Subsequent detachment and elevation of the Schneiderian membrane at the sinus floor significantly reduced the perforation site, and bone grafting with implant placement was performed simultaneously. This alleviated the need to surgically repair the perforation. The lateral opening was either uncovered or repositioned using bony window lids. Healing abutments were connected after six months, and the final prosthesis was placed after two months. At the 1-year follow-up, the antral pseudocysts had resolved with no specific recurrence, and the stability of the augmented sinus was maintained with excellent implant survival. Within the limitations of our findings, the “one-bony-window” technique can be suggested for the simultaneous removal of large antral pseudocysts and maxillary sinus floor augmentation with favorable clinical outcomes.

## 1. Introduction

Maxillary sinus floor augmentation (MSFA) using the lateral window approach involves elevation of the Schneiderian membrane through an access opening in the maxillary sinus lateral wall and creates confined space underneath the elevated membrane filled with grafting materials to increase limited residual ridge height [1,2]. A predictable long-term outcome regarding implant survival in MSFA has been reported [3]. However, risks of intraoperative and postoperative complications may occur related to the lateral window approach, and perforation of the Schneiderian membrane is the most frequent complication during the procedure [4]. Different treatment strategies including post-perforation clot formation, placement of collagen membrane, suturing, and use of platelet-rich fibrin are introduced to manage the membrane perforation [4,5,6]. Other complications such as wound infection, post-operative sinusitis, and graft failure may also be involved with MSFA, although the incidence is relatively low [4]. In the presence of maxillary sinus pathologies, the potential risks of complications are increased; therefore, an accurate diagnosis and careful clinical and radiographic assessments before surgery are critical [7].

Maxillary sinus cysts are frequently diagnosed on radiographs. These cysts are classified into mucoceles, postoperative maxillary cysts, mucous retention cysts, and pseudocysts, with different clinicopathologic aspects [8]. Mucous retention cysts and antral pseudocysts are among the most common lesions of the maxillary sinus [9]. Mucous retention cysts are fluid-filled masses with thin epithelial linings that result from the obstruction of seromucous glands, and several etiologies including trauma from tooth extraction, dental infection, and allergies are suggested to be involved with the occurrence of the lesions [10]. On the other hand, pseudocysts lack epithelial walls due to diffuse subepithelial accumulation of inflammatory exudate and have been associated with allergies and respiratory and viral infections, although the etiologies are yet to be confirmed [11]. Antral pseudocysts usually appear as faint and dome-shaped radiopaque lesions with homogenous density [12]. It is an asymptomatic benign lesion that does not require treatment under normal circumstances. However, the presence of large antral pseudocysts may increase the risk of ostial obstruction and dysventilation following upward shift of the lesions by sinus lift [13]. Moreover, preoperative sinus pathology may interfere with Schneiderian membrane mobilization and increase the risk of membrane perforation, which can contaminate and infect graft materials with the leakage of cystic fluid or purulent exudate [14,15].

Conventionally, antral cystic lesions are usually treated before MSFA, and Caldwell–Luc surgery was considered the gold standard for complete removal of the lesion from maxillary sinus, which is replaced with new mucosa [16,17]. However, Caldwell–Luc surgery is associated with increased postoperative surgical trauma, patient morbidity, and longer healing periods. Endoscopic sinus surgery can also be considered in the treatment of sinonasal impairments. Endoscopic-assisted MSFA through the lateral maxillary wall allows for a direct and clear visualization of the surgical field without preparation of huge or multiple bone windows, which helps to reduce the risk of membrane perforation during sinus lift [18]. It also allows for a one-stage procedure for MSFA combined with the removal of maxillary sinus pathologies [19]. Despite the advantages, it is not easy to access endoscopic surgery due to the need for special instruments or settings accompanied by medical assistance.

Recently, technical surgical modifications to simultaneously remove antral pseudocysts and perform MSFA using either the one- or two-stage approach have been introduced into dentistry [20,21,22]. Chiapasco et al. described the formation of an additional small round bony access above the sinus window opening for enucleation of pseudocyst while preserving the integrity of the Schneiderian membrane periosteal layer during the simultaneous approach [20]. Yu et al. prepared a bony window (a smaller rounded opening) to first remove the pseudocyst, and then, created a larger bony window surrounding the former to elevate the sinus membrane [21,22]. Previous studies have utilized a smaller size of bony window during the first surgical step to remove a cyst following the aspiration of mucous fluid, which may be advantageous in minimizing damage to the sinus mucosa by limiting the membrane perforation. However, small windows make it difficult to access instruments and remove large cysts without rupture. Leakage of inflammatory exudate from disintegrated lesion can increase the risk of graft contamination when one-stage MSFA is planned simultaneously with the cyst removal.

In this case series, we describe the “one-bony-window” approach, which is a technical surgical modification of the previous one-stage approach, for simultaneous cyst removal and MSFA in four patients. It includes the creation of a large-sized lateral window opening and mesiodistally long intentional incision on the upper part of the Schneiderian membrane to facilitate cyst removal without deformation or leakage from the rupture. The purpose of this article is to present a one-bony-window approach for favorable clinical outcomes in the removal of a large antral pseudocyst and one-stage MSFA.

## 2. Case Presentation

This case series includes four patients who visited a private practice in periodontics and implant dentistry. All the patients presented with a limited residual bone height in the posterior maxilla that would benefit from sinus lifting. Moreover, a large antral pseudocyst was present in each patient. To simultaneously remove the antral cyst and perform MSFA, we modified the lateral window opening technique to develop the “one-bony-window approach”. Briefly, a mucoperiosteal flap with vertical incisions in the posterior maxilla was fully reflected and an oval-shaped lateral access window opening, measuring approximately 15 mm × 20 mm, was prepared. After exposing the Schneiderian membrane of the lateral wall, a long horizontal incision was made in the upper part of the membrane using a #15 Bard-Parker blade. The antral pseudocyst was removed through the incised hole within the membrane using a Pincette or tissue plier. Subsequently, membrane elevation of the sinus floor, which should not be performed prior to cyst removal, was carried out carefully to avoid membrane perforation and to secure the space for bone graft materials. The incised membrane in the upper part was left without additional repair. The graft materials for MSFA were deposited into the confined space beneath the membrane elevation, and dental implants were placed simultaneously. The lateral opening was either repositioned with the bony window removed or left uncovered. Finally, the mucoperiosteal flap was sutured to facilitate primary closure.

### 2.1. Case 1

**Figure 1:**
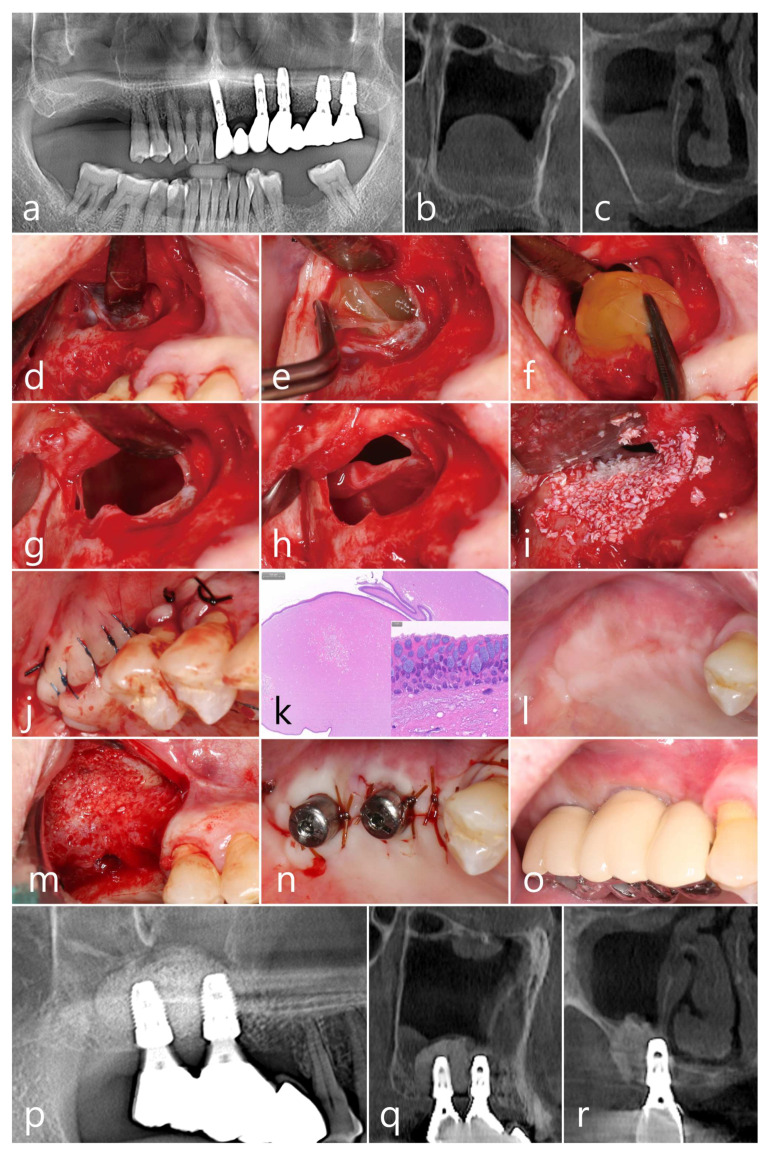
Case 1. Preoperative radiographs. A dome-shaped antral pseudocyst in the right maxillary sinus is shown in the panoramic (**a**), sagittal (**b**), and coronal (**c**) CBCT images. The “one-bony-window” technique including preparation of an elliptical lateral sinus access opening (**d**), intentional perforation at the upper part of the exposed Schneiderian membrane (**e**), removal of the antral pseudocyst with a large mucosal perforation site (**f**,**g**), detachment and elevation of sinus floor membrane with significant reduction in the perforation size (**h**), sinus floor augmentation with graft materials and implant placements (**i**), and flap closure (**j**). Histological features of the antral pseudocyst specimens (**k**). The clinical appearance at 6 months of follow-up (**l**). Surgical re-entry and healing abutment connection (**m**,**n**). Post-prosthetic delivery (**o**). Postoperative radiographic features of the augmented sinus floor in the panoramic (**p**), sagittal (**q**), and coronal (**r**) CBCT images, taken 1 year after prosthetic loading.

A 67-year-old male smoker was scheduled for dental implant placement in the maxillary right posterior edentulous ridge. The residual bone height was limited to within 3 mm, due to the alveolar ridge resorption and a pneumatized sinus floor (Figure 1a). A preoperative CBCT (sagittal and coronal views) showed a large dome-shaped antral cyst (20.5 mm × 27.8 mm) in the right maxillary sinus, which occupied half of the sinus cavity (Figure 1b,c). After mucoperiosteal flap reflection, a single bony lateral window was prepared using a rotary round bur, and the bony lid was removed (Figure 1d). Part of the mucous membrane was incised by intentionally perforating the upper Schneiderian membrane, exposing a yellowish cystic wall (Figure 1e). Using a pair of tissue pliers, the antral pseudocyst was removed through the perforated membrane. The original shape of the cyst was preserved and there was no rupture of the cystic wall or outflow of cystic fluid (Figure 1f). A large perforation was observed (Figure 1g), and the remaining membrane attached to the sinus floor was carefully separated using sinus elevation instruments (Genoss, Suwon, Republic of Korea). By elevating the membrane of the sinus floor, the size of the perforated area was greatly reduced. This reduction is due to the perforated area moving further to the buccal side, with a reduction in tension applied to the mucous membrane and mucosal folding (Figure 1h). The sinus floor was augmented with xenografts (OsteonTM Xeno; Genoss, Suwon, Republic of Korea). Additional repair of the perforated membrane was not necessary (Figure 1i). Two Ø 4.8 mm × 10 mm SLA (sandblasts, large grit, acid-etched) implants (Implantium, Dentium, Suwon, Republic of Korea) were simultaneously placed and submerged (Figure 1j). Postoperatively, systemic antibiotics (ciprofloxacin 500 mg; Ildong Pharmaceutical Co., Seoul, Republic of Korea) and nonsteroidal anti-inflammatory drugs (etodolac; Yuhan Pharmaceutical Co., Seoul, Republic of Korea) were prescribed for 7 days. To avoid increasing pressure within the sinus, the patient was advised not to blow his nose. Histological examination of the resected specimen demonstrated an inflamed maxillary sinus lining surrounding an inner fibrous connective tissue, which was diagnosed as an antral pseudocyst (Figure 1k). The patient had transient nasal bleeding, facial swelling, and hematoma, but healing was uneventful (Figure 1l). Surgical re-entry and abutment connection were performed after six months (Figure 1m,n). Regenerated hard tissue covering the lateral window site was observed. Prosthetic loading was performed 2 months after healing (Figure 1o). At the 1-year follow-up, a dome-shaped augmentation of the graft volume surrounding the implant fixtures was observed in the panoramic view (Figure 1p). Additionally, resolved cystic lesions adjacent to the well-defined grafted borders were observed on sagittal and coronal CBCT images (Figure 1q,r).

### 2.2. Case 2

**Figure 2:**
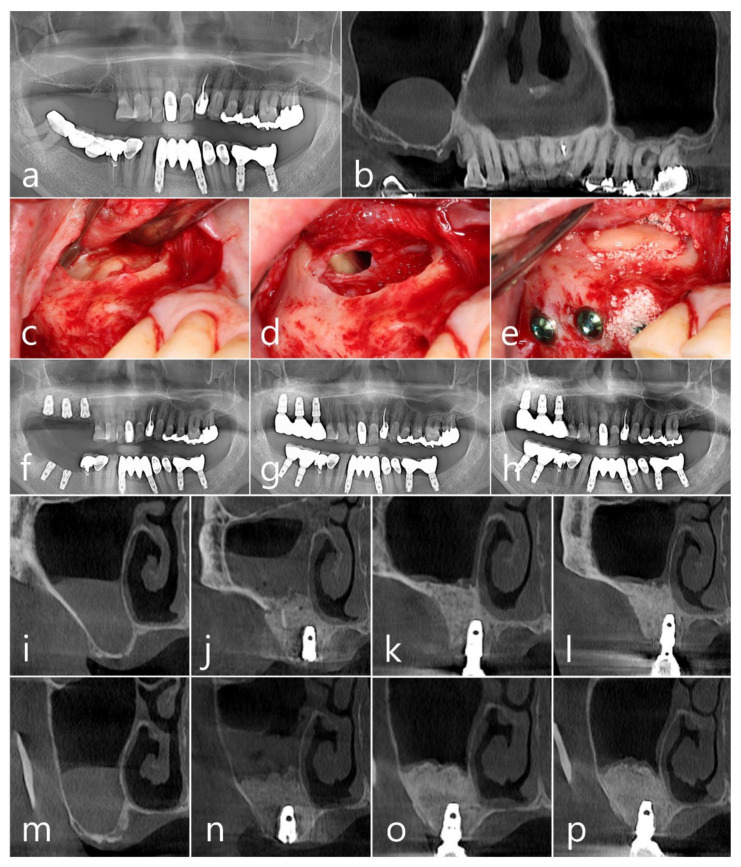
Case 2. Preoperative radiographs. A dome-shaped antral pseudocyst in the right maxillary sinus is shown in the panoramic radiograph (**a**) and CBCT (**b**). The “one-bony-window” technique showing lateral sinus window opening (**c**), intentional perforation at the upper part of the Schneiderian membrane and removal of antral pseudocyst (**d**), and sinus floor membrane elevation following bone grafting with implant placements, with repositioning of bony window (**e**). Panoramic view taken immediately after sinus floor augmentation and implant placement (**f**), after prosthetic delivery (**g**), and after 1 year of loading (**h**). Coronal CBCT images of the right maxillary first molar site before surgery (**i**), immediately after surgery (**j**), after prosthetic delivery (**k**), and after 1 year of loading (**l**). Coronal CBCT images of the right maxillary second molar site in the corresponding periods are described above (**m**–**p**).

A 72-year-old male non-smoker, who was taking antihypertensive drugs, presented for consultation regarding a faint dome-shaped lesion in the right maxillary sinus (Figure 2a). The panoramic CBCT view demonstrated a well-delineated homogenous lesion, measuring approximately 22.5 mm × 22.7 mm, on the sinus floor (Figure 2b). The lesion was removed using the one-bony-window technique described in Case 1 (Figure 2c,d). However, for this patient, the removed bony window was repositioned at the opening. Synthetic biphasic calcium phosphate (OsteonTM II; Genoss, Suwon, Republic of Korea) was used as a bone graft substitute (Figure 2e). One Ø 4.3 mm × 10 mm and two Ø 4.8 mm × 10 mm SLA-surface implants (Implantium, Dentium, Suwon, Republic of Korea) were placed. The implants were submerged, and the patient received the same prescription and postoperative precautions as in Case 1. The patient experienced postoperative pain, swelling, nasal bleeding, and hematoma. Healing was uneventful. The healing abutment was connected after 6 months, and prosthetic loading was performed 2 months after uncovering the abutments. In the post-surgical panoramic radiographs (Figure 2f) (taken at prosthesis delivery (Figure 2g) and 1 year of loading (Figure 2h), respectively), no specific crestal bone loss around the implants was observed, and the augmented sinus floor showed no significant decrease. In the coronal CBCT images obtained at the first molar site (Figure 2i–l) and second molar site (Figure 2m–p), taken at each procedure corresponding to the panoramic view, no specific leakage of the bone graft material, or recurrence of the antral pseudocyst, was evident.

### 2.3. Case 3

**Figure 3:**
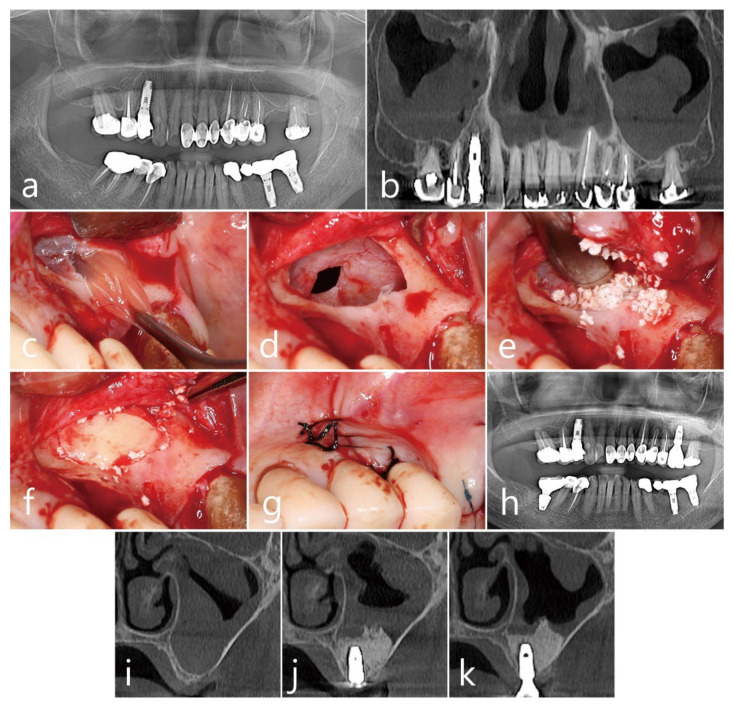
Case 3. Preoperative radiographic features of both maxillary sinuses are shown in the panoramic (**a**) and CBCT (**b**) images. The “one-bony-window” technique in left maxillary sinus showing lateral sinus window opening and removal of antral pseudocyst via intentional perforation site (**c**), sinus floor membrane elevation and reduced size of perforation (**d**), sinus bone grafting with implant placements (**e**), repositioning of bony window (**f**), and primary flap closure (**g**). Panoramic view obtained immediately after sinus augmentation and implant placement (**h**). Coronal CBCT images before surgery (**i**), immediately after surgery (**j**), and after 1 year of loading (**k**).

A 79-year-old female non-smoker visited the clinic for implant placement at the left maxillary first molar site. The site had limited residual bone height and a large dome-shaped cystic appearance, measuring 21.2 mm × 30.4 mm, in the left maxillary sinus cavity (Figure 3a,b). After removing the cyst and lifting the sinus floor using the one-bony-window technique (Figure 3c,d), xenograft bone augmentation (OsteonTM Xeno, Genoss, Suwon, Republic of Korea) was performed simultaneously with the placement of one Ø 4.8 mm × 10 mm SLA-surface implant (Implantium, Dentium, Suwon, Republic of Korea). Similar to Case 2, we repositioned the lateral bony window (Figure 3f), and then submerged the implant (Figure 1g). During early healing, a partial wound dehiscence developed at the incision line, and the patient experienced nasal bleeding and hematoma. Subsequently, healing occurred without further complications. The abutments were uncovered 6 months after MSA, and the prosthesis was placed 2 months later (Figure 3h). Preoperative coronal CBCT images showed a large antral pseudocyst (Figure 3i) and traces of graft leakage at the intentionally perforated site after surgery (Figure 3j). However, the leak was limited and did not change the augmented volume significantly. At the 1-year follow-up, recurrence of the antral cyst was not evident (Figure 3k).

### 2.4. Case 4

**Figure 4:**
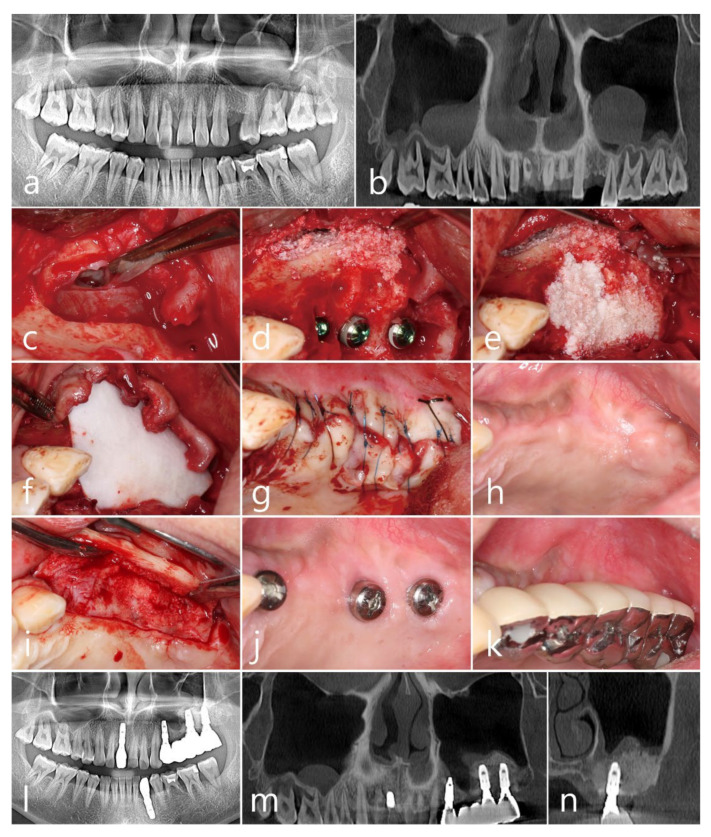
Case 4. Preoperative radiographs. Dome-shaped antral pseudocysts in both maxillary sinuses are shown in the panoramic (**a**) and CBCT (**b**) images. The “one-bony-window” technique including the preparation of an elliptical lateral window opening at the left maxilla and the removal of the antral pseudocyst through intentional perforation (**c**), sinus bone augmentation with implant placement (**d**), additional bone graft for peri-implant bone defects covered by a resorbable collagen membrane (**e**,**f**), and flap closure (**g**). The clinical appearance after 6 months of healing (**h**), at surgical reentry (**i**), connection of healing abutments (**j**), and prosthetic delivery (**k**). Panoramic (**l**) and CBCT (**m**,**n**) images at the 1-year follow-up.

A 42-year-old male smoker visited the clinic because of severe mobility of the posterior teeth in both the right and left maxilla. Preoperative panoramic radiography and CBCT revealed severe periodontal loss at the root apex of both maxillary molars (Figure 4a). Additionally, a dome-shaped antral pseudocyst, measuring 20.5 mm × 25.3 mm, attached to the sinus floor was observed in the sinus cavities bilaterally (Figure 4b). Replacement with an implant-supported fixed bridge from the first premolar to the second molar of the left maxilla was planned to be carried out first. After extraction of the left maxillary second premolar and first and second molars, MSFA was immediately performed using the one-bony-window technique with an elliptical lateral sinus window. The Schneiderian membrane was exposed without tears. A horizontal incision was made at the top of the window to access the cyst in the sinus, and the antral pseudocyst was removed in its entirety using tissue pliers (Figure 4c). After the detachment and elevation of the Schneiderian membrane from the floor, xenograft bone augmentation (OsteonTM Xeno, Genoss, Suwon, Republic of Korea) and implant placement (Implantium, Dentium, Suwon, Republic of Korea; one Ø 3.8 mm × 10 mm and two Ø 4.3 mm × 10 mm implants) were performed (Figure 4d). The peri-implant defects were grafted, covered with a resorbable collagen membrane (Genoss, Suwon, Republic of Korea) (Figure 4e,f), submerged, and closed with a primary flap (Figure 4g). After surgery, the patient experienced severe facial swelling, hematoma, and pain. The surgical site healed uneventfully (Figure 4h). The abutments were uncovered six months after surgery, which showed regenerated bone at the peri-implant defects (Figure 4i,j). The final prosthesis was placed two months later (Figure 4k). Panoramic radiography and CBCT taken after 1 year of prosthetic loading showed a well-maintained volume of augmented bone, with no cystic lesions observed (Figure 4l–n).

## 3. Discussion

The one-stage approach using the one-bony-window technique in the presence of a large antral pseudocyst resulted in successful cyst removal and simultaneous MSFA without graft contamination or postoperative sinus infection. Early postoperative complications, such as nasal bleeding, swelling, and hematoma, occurred. Subsequently, healing occurred with no further clinical problems. Cyst recurrence or graft failure was not observed during the follow-up period, and all implants were successfully maintained.

Schneiderian membrane perforation is the most common accidental intraoperative complication during sinus lift [23,24]. Factors, such as thickness and morphology of the Schneiderian membrane, anatomy of the sinus, limited residual ridge height, and sinus pathology, can increase the risk. Sinus pathology may be accompanied by poor membrane vascularity and elasticity and reduced membrane resistance to elevation [15,24]. Membrane perforation in the presence of sinus pathoses may also lead to graft contamination and sinus infection with the leakage of cystic fluid or inflammatory exudate unless the lesion is enucleated ahead of MSFA. Management of the preexisting maxillary lesions should be carefully planned.

The size and location of the lesion may influence the timing of MSFA (either immediately after the removal of antral pseudocysts or after delayed healing for several months) [12,25]. For small pseudocysts, MSFA can be performed without removal or other treatment [26]. However, large antral pseudocysts are likely to block osteomeatal complex patency, which can cause postoperative sinusitis or retention cysts and perforation during sinus lift [19]. Aspiration of cystic fluid can compress the volume and simplify removal. However, treatment confined to mucus aspiration showed recurrence in 30% of maxillary cysts, due to residual connective tissue from the pseudocyst [21,27]. The two-stage approach is relatively problem-free, especially issues related to leakage and graft displacement. However, it requires a postoperative healing period of at least 3 months and additional surgeries thereafter. Difficulties in elevation of the Schneiderian membrane due to scar tissues during the first access may also occur.

In a recent report comparing one- and two-stage interventions for cyst removal and MSFA, both approaches showed comparable histological results and clinical outcomes, including implant survival, marginal bone resorption, and complication rates [22]. Postoperative complications, such as infection, were also infrequent in both groups. However, the one-stage approach was technically more challenging. Previous studies have described the enucleation of an antral pseudocyst through a smaller bony access prepared additionally or prior to an enlarged bony window for sinus membrane elevation [20,21,22]. The purpose was to avoid excessive damage to the sinus mucosa adjacent to the intentional perforation and to minimize progressive tearing, but the access to the lesion could still be limited. The one-bony-window technique used in the present study involved the preparation of a large bony opening to enucleate the pseudocyst, with a relatively long incised intentional perforation in the upper portion. The wide access facilitates cyst removal without damaging the original shape of the lesion. The risk of graft contamination can also be reduced especially when the cystic wall is vulnerable and likely to rupture even with light manipulation.

In severe cases, Schneiderian membrane perforation may result in mucosal thickening, displacement of graft particles, and postoperative maxillary sinusitis. Some studies have reported an association between membrane perforation and adverse effects, compromising sinus floor augmentation and implant survival [28,29]. On the other hand, several studies have supported the conflicting opinion that membrane perforation is not associated with postoperative infection or implant failure, probably because of improved access for instruments and materials, and repair techniques [23,26,30]. Proper strategies to manage a perforated membrane are suggested to reduce complications [6,24,31]. Currently, intentional perforation during sinus lift is mostly indicated for the removal of implants displaced into the sinus cavity [32] and for treating sinus mucosal thickening or pathology. It is assumed that the procedure may help manage minor pathologic lesions, such as antral pseudocysts.

The proposal to create an intentional long incised perforation in the Schneiderian membrane as required in the one-bony-window technique can be counter-intuitive to clinicians as the efforts to block perforation are pursed. Furthermore, the perforated membrane was not covered by some collagen membrane or sutured. Instead, careful detachment and elevation of the Schneiderian membrane at sinus floor distant from the intentional perforation relieved the tension on the sinus mucosa, reduced the size of the perforation, and moved the position further buccally with mucosal folding. The whole procedure could be easily performed with good visual access through a large-sized window. Interestingly, a previous study by Park et al. reported that a non-repaired perforation of the Schneiderian membrane did not adversely affect the clinical and radiographic outcomes of MSFA [15]. In the study, a Prichard elevator was inserted into the sinus cavity to prevent displacement of bone graft material, and the material was condensed only in the direction of the sinus floor. Successful bone grafting was possible without notable leakage of the bone graft material into the perforated mucosa that had not been repaired, and the leakage of bone graft particles was naturally drained by mucociliary clearance and did not stagnate in the maxillary sinus [33]. In the present cases, although the perforation site was not repaired with an additional collagen membrane and was not completely covered, the collapsed perforation, with its size and buccally displaced location after sinus lift, was compatible with stabilizing the space and minimizing contact with the graft materials.

Implant survival and ossification of sinus bone grafts may be enhanced when the lateral access opening sites are covered with barrier membranes or repositioned using a bony window [34,35]. However, controversial opinions suggest that there are no significant differences in implant treatment outcomes after MSFA, with or without coverage of the lateral windows, although a higher percentage of newly formed bone was detected in the coverage group [36]. Cases in this study were treated with either repositioning of the lateral bony window or a lateral opening without coverage, and the clinical and radiographic outcomes were comparable and consistent.

Despite the favorable outcomes in implant survival, maintenance of augmented bone height, and no recurrence of cystic lesions, the findings are limited in small-sized samples with short-term observation periods. To verify our results, controlled prospective studies with larger sample sizes should be conducted in the future.

## 4. Conclusions

Despite the limitations present in the case series of four patients with large maxillary antral pseudocysts, the “one-bony-window” technique can be utilized in simultaneous cyst removal and sinus floor augmentation and showed favorable outcomes of implant survival and well-maintained augmented bone height in short-term periods.

## Data Availability

Data are contained within the article.

## References

[1] Boyne P.J., James R.A. (1980). Grafting of the maxillary sinus floor with autogenous marrow and bone. J. Oral Surg..

[2] Tatum H.J. (1986). Maxillary and sinus implant reconstructions. Dent. Clin. N. Am..

[3] Raghoebar G.M., Onclin P., Boven G.C., Vissink A., Meijer H.J.A. (2019). Long-term effectiveness of maxillary sinus floor augmentation: A systematic review and meta-analysis. J. Clin. Periodontol..

[4] Hsu Y.T., Rosen P.S., Choksi K., Shih M.C., Ninneman S., Lee C.T. (2022). Complications of sinus floor elevation procedure and management strategies: A systematic review. Clin. Implant Dent. Relat. Res..

[5] Park W.B., Kang K.L., Han J.Y. (2019). Factors influencing long-term survival rates of implants placed simultaneously with lateral maxillary sinus floor augmentation: A 6- to 20-year retrospective study. Clin. Oral Implants Res..

[6] Díaz-Olivares L.A., Brinkmann J.C.B., Martínez-Rodríguez N., Martínez-González J.M., López-Quiles J., Leco-Berrocal I., Meniz-García C. (2021). Management of Schneiderian membrane perforations during maxillary sinus floor augmentation with lateral approach in relation to subsequent implant survival rates: A systematic review and meta-analysis. Int. J. Implant Dent..

[7] Kim S.W., Lee I.H., Kim S.W., Kim D.H. (2019). Points to consider before the insertion of maxillary implants: The otolaryngologist’s perspective. J. Periodontal Implant. Sci..

[8] Beaumont C., Zafiropoulos G.G.G., Rohmann K., Tatakis D.N. (2005). Prevalence of maxillary sinus disease and abnormalities in patients scheduled for sinus lift procedures. J. Periodontol..

[9] Meer S., Altini M. (2006). Cysts and pseudocysts of the maxillary antrum revisited. J. South Afr. Dent. Assoc..

[10] Anitua E., Alkhraisat M., Torre A., Eguia A. (2021). Are mucous retention cysts and pseudocysts in the maxillary sinus a risk factor for dental implants? A systematic review. Med. Oral Patol. Oral Cir. Bucal..

[11] Mardinger O., Manor I., Mijiritsky E., Hirshberg A. (2007). Maxillary sinus augmentation in the presence of antral pseudocyst: A clinical approach. Oral Surg. Oral Med. Oral Pathol. Oral Radiol. Endod..

[12] Kara I.M., Küçük D., Polat S. (2010). Experience of maxillary sinus floor augmentation in the presence of antral pseudocysts. J. Oral Maxillofac. Surg..

[13] Kara M.I., Kirmali O.M.I., Kirmali O., Ay S. (2012). Clinical evaluation of lateral and osteotome techniques for sinus floor elevation in the presence of an antral pseudocyst. Int. J. Oral Maxillofac. Implants.

[14] Pikos M.A. (2008). Maxillary sinus membrane repair: Update on technique for large and complete perforations. Implant Dent..

[15] Park W.B., Han J.Y., Kang P., Momen-Heravi F. (2019). The clinical and radiographic outcomes of Schneiderian membrane perforation without repair in sinus elevation surgery. Clin. Implant Dent. Relat. Res..

[16] Datta R.K., Viswanatha B., Harsha M.S. (2016). Caldwell Luc Surgery: Revisited. Indian J. Otolaryngol. Head Neck Surg..

[17] Lin Y., Hu X., Metzmacher A.R., Luo H., Heberer S., Nelson K. (2010). Maxillary sinus augmentation following removal of a maxillary sinus pseudocyst after a shortened healing period. J. Oral Maxillofac. Surg..

[18] Giovannetti F., Raponi I., Priore P., Macciocchi A., Barbera G., Valentini V. (2019). Minimally-Invasive Endoscopic-Assisted Sinus Augmentation. J. Craniofac. Surg..

[19] Abu-Ghanem S., Kleinman S., Horowitz G., Balaban S., Reiser V., Koren I. (2015). Combined maxillary sinus floor elevation and endonasal endoscopic sinus surgery for coexisting inflammatory sinonasal pathologies: A one-stage double-team procedure. Clin. Oral Implants Res..

[20] Chiapasco M., Palombo D. (2015). Sinus grafting and simultaneous removal of large antral pseudocysts of the maxillary sinus with a micro-invasive intraoral access. Int. J. Oral Maxillofac. Surg..

[21] Yu H., Qiu L. (2019). Histological and clinical outcomes of lateral sinus floor elevation with simultaneous removal of maxillary sinus pseudocysts. Clin. Implant Dent. Relat. Res..

[22] Yu H., Tang Y., He D., Qiu L. (2023). Immediately or delayed sinus augmentation after pseudocyst removal: A randomized trial. Clin. Implant Dent. Relat. Res..

[23] Testori T., Weinstein T., Taschieri S., Wallace S.S. (2019). Risk factors in lateral window sinus elevation surgery. Periodontol. 2000.

[24] Testori T., Tavelli L., Scaini R., Saibene A.M., Felisati G., Barootchi S., Decker A.M., Deflorian M.A., Rosano G., Wallace S. (2023). How to avoid intraoperative and postoperative complications in maxillary sinus elevation. Periodontol. 2000.

[25] Bhattacharyya N. (2000). Do maxillary sinus retention cysts reflect obstructive sinus phenomena?. Arch. Otolaryngol. Head Neck Surg..

[26] Timmenga N.M., Raghoebar G.M., van Weissenbruch R., Vissink A. (2003). Maxillary sinus floor elevation surgery. A clinical, radiographic and endoscopic evaluation. Clin. Oral Implants Res..

[27] Wang J.H., Jang Y.J., Lee B.J. (2007). Natural course of retention cysts of the maxillary sinus: Long-term follow-up results. Laryngoscope.

[28] Hernandez-Alfaro F., Torradeflot M.M., Marti C. (2008). Prevalence and management of Schneiderian membrane perforations during sinus-lift procedures. Clin. Oral Implants Res..

[29] Tükel H.C., Tatli U. (2018). Risk factors and clinical outcomes of sinus membrane perforation during lateral window sinus lifting: Analysis of 120 patients. Int. J. Oral Maxillofac. Surg..

[30] Becker S.T., Terheyden H., Steinriede A., Behrens E., Springer I., Wiltfang J. (2008). Prospective observation of 41 perforations of the Schneiderian membrane during sinus floor elevation. Clin. Oral Implants Res..

[31] Park W.B., Kim J.K., Kim Y.J., Kang P., Lim H.C., Han J.Y. (2023). Changes in sinus mucosal thickening in the course of tooth extraction and lateral sinus augmentation with surgical drainage: A cone-beam computed tomographic study. Clin. Oral Implants Res..

[32] An J.H., Park S.H., Han J.J., Jung S., Kook M.S., Park H.J., Oh H.K. (2017). Treatment of dental implant displacement into the maxillary sinus. Maxillofac. Plast. Reconstr. Surg..

[33] Park W.B., Cho N.J., Kang P. (2022). Tomographic Imaging of Mucociliary Clearance Following Maxillary Sinus Augmentation: A Case Series. Medicina.

[34] Wang W.B., Zhang J., Ren L., Yang G. (2022). Repositioning of the bone window in lateral sinus floor elevation with simultaneous implant placement: A retrospective radiographic study. Clin. Oral Implants Res..

[35] Tawil G., Tawil P., Khairallah A. (2016). Sinus Floor Elevation Using the Lateral Approach and Bone Window Repositioning I: Clinical and Radiographic Results in 102 Consecutively Treated Patients Followed from 1 to 5 Years. Int. J. Oral Maxillofac. Implants.

[36] Starch-Jensen T., Deluiz D., Duch K., Tinoco E.M.B. (2019). Maxillary Sinus Floor Augmentation with or without Barrier Membrane Coverage of the Lateral Window: A Systematic Review and Meta-Analysis. J. Oral Maxillofac. Res..

